# Radiological and radiomic outcomes following pressure-controlled fibrinolytic irrigation therapy for acute subdural hematoma

**DOI:** 10.1016/j.bas.2026.107719

**Published:** 2026-11-02

**Authors:** Marco Bissolo, Roberto Doria-Medina, Mazin Omer, Mukesch Johannes Shah, Manou Overstijns, Theo Demerath, Lucas Becker, Eva Rohr, Klaus-Jürgen Buttler, Florian Volz, Jürgen Beck, Roland Roelz

**Affiliations:** a Department of Neurosurgery, Medical Center - University of Freiburg, Faculty of Medicine, University of Freiburg, Freiburg, Germany; b Department of Neuroradiology, Medical Center - University of Freiburg, Faculty of Medicine, University of Freiburg, Freiburg, Germany

**Keywords:** Acute subdural hematoma, Twist-drill craniostomy, Fibrinolytic irrigation, Radiomics, Minimally invasive neurosurgery, Hematoma liquefaction

## Abstract

**Introduction:**

The optimal management of neurologically stable acute subdural hematoma (aSDH) remains uncertain. Twist-drill-craniostomy with pressure-controlled-fibrinolytic irrigation (TDC-FIT) offers a minimally invasive approach, but its imaging effects remain poorly characterized.

**Research question:**

To assess radiological, radiomic, and clinical outcomes after TDC-FIT.

**Material and methods:**

This retrospective, single-center study included neurologically stable aSDH patients treated between July 2023 and June 2025. Pretreatment computed tomography (CT) scans and the first CT before irrigation discontinuation were assessed for hematoma volume, midline shift, attenuation in Hounsfield units (HU), skewness, and kurtosis. Paired nonparametric tests compared imaging measurements. Clinical outcomes, complications, and modified Rankin Scale (mRS) scores were recorded.

**Results:**

Twenty-five patients were included (52% male; mean age, 83 years). Median hematoma volume decreased from 83.6 cm^3^ (IQR 62.4–111.0) to 21.0 cm^3^ (IQR 5.0–35.6; p < 0.0001), and midline shift from 6.0 to 0.0 mm (p = 0.00065). Median percentage volume reduction was 74.9% (IQR 63.3–93.1%). Mean intralesional attenuation decreased from a cohort median of 59.9 to 48.0 HU (p = 0.0025); skewness changed from −0.56 to −0.14 (p = 0.019). Median mRS was 2 premorbidly, 3 at discharge, and 2 at 3 months. Procedure-related complications comprised rebleeding requiring craniotomy in two patients (8%), symptomatic chronic SDH recurrence in two (8%), and device failure in one (4%). Both rebleeding scans preceded rescue craniotomy.

**Discussion and conclusion:**

TDC-FIT was associated with hematoma reduction and changes in attenuation and skewness. These findings may reflect altered hematoma composition, but their biological significance remains uncertain. Prospective controlled studies are needed to establish safety and efficacy.

## Highlights

• **What is already known on this topic.**Evidence for minimally invasive treatment of neurologically stable acute subdural hematoma is limited, particularly regarding radiological and radiomic outcomes.
• **What this study adds**TDC-FIT achieved marked hematoma and midline shift reduction with radiomic changes consistent with clot liquefaction and acceptable complication rates.
• **How this study might affect research, practice or policy**These findings support prospective evaluation of TDC-FIT as a minimally invasive treatment option.

## 1 Introduction

Management of neurologically stable acute subdural hematoma (aSDH) remains a topic of ongoing debate. Large multicenter registries, including CENTER-TBI and TRACK-TBI, show that essentially all patients worldwide are managed either conservatively or with early craniotomy, despite comparable injury severity and similar functional outcomes across centers (van Erp et al., 2023; Van Essen et al., 2025). This binary approach reflects persistent uncertainty in clinical decision-making, particularly for elderly, multimorbid patients with stable hematomas and preserved neurological function, where immediate craniotomy may be unnecessary and carries inherent surgical risks (Bajsarowicz et al., 2015).

Emerging evidence suggests that extravasated blood in the subdural space is biologically active rather than a purely passive mass (Jussen et al., 2017). Blood breakdown products can induce oxidative stress, inflammation, and neuronal injury, and experimental studies indicate that subdural blood may disrupt the blood–brain barrier and contribute to neurological decline in addition to mass effect (Jussen et al., 2017). These observations underscore the potential value of interventions that remove hematoma in a minimally invasive manner and gradually, rather than relying solely on conservative management or evacuation via large craniotomy (Jussen et al., 2017; Cohen et al., 2004).

In this context, twist-drill craniostomy with pressure-controlled fibrinolytic irrigation (TDC-FIT) has emerged as a novel therapeutic option for neurologically stable patients (Bissolo et al., 2025a, 2025b, 2026; Doria-Medina et al., 2025). By providing minimally invasive hematoma evacuation through controlled clot liquefaction and drainage, TDC-FIT may reduce surgical morbidity while effectively decreasing hematoma volume and mass effect (Bissolo et al., 2025a, 2025b). However, quantitative data on hematoma volume dynamics, radiomic characteristics, and associations with functional outcomes are lacking. We therefore conducted a retrospective volumetric and radiomic analysis of stable aSDH patients treated with TDC-FIT to evaluate hematoma response and explore its clinical impact.

## 2 Methods

### 2.1 Study design and patient selection

We conducted a retrospective single-center cohort study of patients with aSDH at a tertiary neurosurgical center between July 1st, 2023, and June 30, 2025. The study was approved by the institutional review board (approval no. 24–1201-S1) and conducted in accordance with the Declaration of Helsinki, with all data anonymized prior to analysis.

### 2.2 Inclusion and exclusion criteria

Patients were considered eligible for minimally invasive management if they were neurologically stable, had a Glasgow Coma Scale (GCS) score of 13–15 and/or presented with neurological symptoms (e.g., aphasia, hemiparesis, seizures, or neglect), and had no indication for immediate surgical evacuation. Additional eligibility criteria included a hematoma thickness of at least 7 mm with evidence of mass effect and maintenance of neurological and radiological stability during an initial 6-h observation period. Radiological stability was assessed by repeat computed tomography (CT) performed 4–6 h after the initial scan and was defined as an increase in hematoma thickness of no more than 2 mm, no increase in midline shift, and no new hemorrhagic lesions or progression of pre-existing intracranial hemorrhage or cerebral contusions.

Patients with concomitant intracerebral hemorrhage were excluded. Neurological deterioration, defined as a worsening GCS score or other clinical deterioration, or radiological progression during the observation period prompted open craniotomy, as clinically indicated. In addition to these predefined clinical and radiological criteria, premorbid functional status and comorbidities were considered on an individualized basis when assessing suitability for minimally invasive treatment, although they were not formal eligibility criteria. Comorbidity burden was assessed using the age-adjusted Charlson Comorbidity Index (aCCI) to provide a more comprehensive characterization of the study population.

### 2.3 Procedure

TDC-FIT was performed at the bedside under local anesthesia, as previously described (Bissolo et al., 2025a, 2025b, 2026; Doria-Medina et al., 2025). Briefly, a 3.5-mm twist-drill craniostomy was created at the frontal or parietal eminence, followed by placement of a double-lumen subdural catheter. The catheter was connected to a closed, pressure-controlled irrigation–drainage system (IRRAflow®, IRRAS AB, Stockholm, Sweden, or LiquoGuard®7, Möller Medical GmbH, Fulda, Germany)(Bissolo et al., 2025a, 2025b, 2026; Doria-Medina et al., 2025)–(Bissolo et al., 2025a, 2025b, 2026; Doria-Medina et al., 2025).

The two systems differed in their irrigation and drainage mechanisms. IRRAflow® combines automated irrigation, cyclical drainage, and continuous intracranial pressure (ICP) monitoring through an integrated dual-lumen catheter. Its control unit regulates irrigation and drainage according to predefined ICP thresholds, automatically interrupting irrigation and switching to drainage-only mode when ICP exceeds the upper limit, or suspending both irrigation and drainage when ICP falls below the lower limit. LiquoGuard® 7, in contrast, enables continuous irrigation with passive drainage and incorporates ICP monitoring with adjustable pressure limits and high- and low-pressure alarms. Its operating pressure range was typically set between −1 and 19 mmHg.

Despite these technical differences, the urokinase concentration and target infusion rate were standardized across both systems. Urokinase was diluted in isotonic solution at concentrations of 100,000 or 200,000 IU/L and administered at a target rate of 50 mL/h, adjusted according to measured ICP and clinical requirements. Intrathecal administration of urokinase constituted off-label use.

Nursing staff received training in the operation and monitoring of both systems and were instructed to promptly report technical issues or abnormal readings. Urokinase solutions were prepared by the medical team. Continuous physician supervision or intermediate-care monitoring was not required solely because of device use, unless warranted by the patient's clinical condition. Infusion rate, treatment duration, cumulative infused volume, and total urokinase dose were prospectively recorded for analysis.

In cases of antithrombotic therapy, treatment was immediately discontinued upon presentation. In patients receiving vitamin K antagonists (VKAs), anticoagulation was reversed using prothrombin complex concentrate (PCC) and vitamin K1. In patients receiving direct oral anticoagulants (DOACs), an initial dose of 3500 IU of PCC was administered, followed by 1000 IU every 12 h for 48 h. Fresh frozen plasma (FFP) was administered in selected cases when coagulation parameters could not be adequately corrected. Surgery was performed only after the institutional coagulation thresholds had been met, defined as a platelet count ≥ 70 × 10^9^/L, an international normalized ratio (INR) < 1.2, and a partial thromboplastin time (PTT) < 38 s. Anti-factor Xa levels were not routinely available and were therefore not used to guide surgical timing. No specific reversal agents targeting factor Xa inhibitors were administered.

### 2.4 Imaging and volumetric analysis

Hematoma segmentation and volumetric analysis were performed using 3D Slicer (v5.8)**,** and radiomic features were extracted with PyRadiomics**.** Hematoma volume (cm^3^), surface area (cm^2^), maximal thickness (mm), and midline shift (mm) at the point of maximal displacement were measured. Three-dimensional hematoma surface area was extracted from the segmented region of interest and calculated from the triangulated surface mesh of the segmentation.

All pre- and post-treatment CT scans were acquired using the same Siemens CT scanner. Before treatment, a follow-up CT scan was performed to confirm hematoma stability. The initial post-treatment CT scan was obtained using the same scanner either when the irrigation fluid appeared clear, with no visible blood, or earlier if a complication occurred, such as rebleeding requiring conversion to open craniotomy. This scan was defined as the first post-treatment imaging assessment.

Voxel-based Hounsfield Unit (HU) features—including mean, median, range, standard deviation, skewness, and kurtosis—were derived from intensity histograms to characterize clot composition. Skewness reflected the asymmetry of HU distributions, while kurtosis described distribution peakedness and dispersion (Dowlatshahi et al., 2011). A shift in skewness toward positive values was interpreted as progressive clot liquefaction, whereas changes in kurtosis indicated homogeneity in clot structure.

### 2.5 Outcomes

We assessed radiological outcomes, including changes in hematoma volume, maximal thickness, and midline shift, as well as radiomic features, including Hounsfield unit (HU)-based metrics, skewness, and kurtosis. Clinical outcomes included modified Rankin Scale (mRS) scores before treatment, at discharge, and at 3-month follow-up, along with reoperations and procedure-related adverse events, including rebleeding, infection, chronic subdural hematoma recurrence, and device malfunction. Comorbidity burden was evaluated using the Age-Adjusted Charlson Comorbidity Index (aCCI). Exploratory analyses were also performed to investigate associations between irrigation parameters and radiological and functional outcomes.

### 2.6 Statistical analysis

Continuous variables are presented as mean ± standard deviation or median with interquartile range (IQR) as appropriate, and categorical variables as counts and percentages. Pre- and post-treatment imaging parameters (hematoma volume, midline shift, thickness, surface area, radiodensity, skewness, and kurtosis) were compared using the Wilcoxon signed-rank test.

Associations between continuous variables were assessed using Spearman's rank correlation coefficient. We analyzed correlations between absolute and percentage hematoma volume reduction and preoperative volume, preoperative median HU, age, irrigation duration, irrigation rate, and fibrinolytic dose. Correlations between modified Rankin Scale scores at 3 months follow-up and the same covariates, including percentage volume reduction, were also examined.

Given the low number of events, complications (rebleeding and recurrence) were analyzed descriptively only, and no multivariable modeling was performed. All tests were two-sided, with a significance threshold of p < 0.05. No adjustment for multiple comparisons was performed due to the exploratory nature of the study; therefore, statistically significant findings should be interpreted cautiously.

## 3 Results

Between July 1st 2023 and June 30th 2025, 28 patients underwent TDC-FIT, of whom 25 met the inclusion criteria. Among the three excluded patients, one required emergent craniotomy before irrigation could be initiated, and two were excluded due to insufficient postoperative CT imaging. Baseline characteristics are summarized in Table 1. Fig. 1 shows the flowchart of patient selection.

**Table 1:** A) Baseline demographic, clinical, and radiological characteristics of the 25 patients with acute subdural hematoma (aSDH) treated with TDC-FIT. Categorical variables are presented as number (percentage), and continuous variables as mean ± standard deviation (range) or median (interquartile range), as appropriate. B) Baseline and post-treatment radiomic characteristics of acute subdural hematomas before and after TDC-FIT. Continuous variables are presented as median (interquartile range).

A)		
***Sex***	male	13 (52.0%)

***Age (years)***		83.1 ± 8.5 (56.0–94.0)

***aCCI***		6 [2–10]

***mRS premorbid***		2 [0–3]

***GCS at admission***		13 [13–14]

***Initial symptoms***		

	Cognitive impairment (GCS = 13)	16 (64.0%)
		
	Headache	4 (16.0%)
	Hemiparesis	4 (16.0%)
	Aphasia	1 (4.0%)

***Anticoagulation***		

	Phenprocoumon	7 (28.0%)
	NOAC	6 (24.0%)
	Antiplatelet	6 (24.0%)

***System***		

	IRRAflow	18 (72.0%)
	LiquoGuard7	7 (28.0%)

***Days to first postop imaging***		5 (4-6)

***Irrigation duration (hours)***		85 (8.0–120.0)

***Infusion rate (ml/h)***		48 (35–76)

***Complication of TDC-FIT***	Overall	5 (20.0%)
	Rebleeding requiring open craniotomy	2 (8%)
	Readmission due to chronic SDH	2 (8%)
	Mechanical failure	1 (4%)
***mRS at discharge***		3 [2–4]

***mRS at last follow-up***		2 [1–3]

B)			
Radiomic parameters	Preoperative	Postoperative	p-value
***Volume (cm*^*3*^*)***	83.78 (62.4-111)	21.0 (5-35.6)	**< 0.001**
***Surface area (cm*^*2*^*)***	28.5 (21.8–35.2)	9.7 (5.3–17.7)	**< 0.001**
***Minimum HU***	−4.0 (−8.0–3.0)	−15.0 (−19.0–-8.0)	**0.0093**
***Maximum HU***	119.0 (105.0–132.0)	107.0 (96.0–129.0)	0.2157
***Kurtosis***	3.46 (2.85–4.1)	3.41 (2.52–4.79)	0.6092
***Skewness***	−0.56 (−0.77–-0.03)	−0.14 (−0.63–0.97)	**0.0203**

**Fig. 1:**
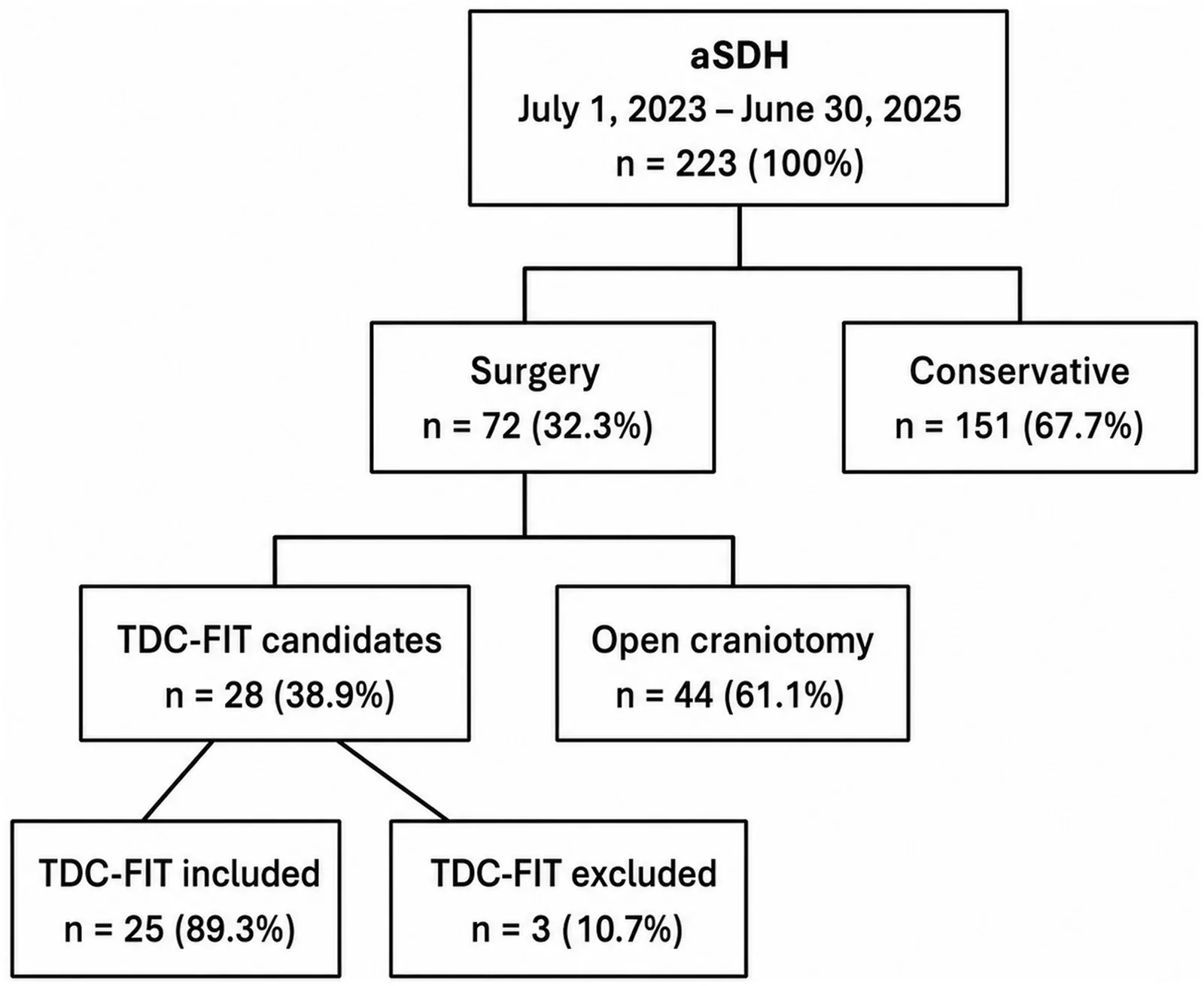
Flowchart of patient selection and subgroup classification for acute subdural hematoma (aSDH) between July 1, 2023, and June 30, 2025 (n = 223). Patients were categorized into surgical (n = 72, 32.3%) and conservative (n = 151, 67.7%) management groups. Within the surgical cohort, 28 patients (38.9%) met the criteria for TDC-FIT, whereas 44 patients (61.1%) underwent open craniotomy. Of the patients eligible for TDC-FIT, 25 (89.3%) underwent the procedure and were included in the study cohort, whereas 3 (10.7%) were excluded from the radiomic analysis because of incomplete imaging data or conversion to emergent craniotomy before irrigation could be initiated.

### 3.1 Irrigation characteristics

IRRAflow was used in 18 patients and LiquoGuard7 in 7. The median duration of irrigation therapy was 85 h (IQR 8.0–120.0). Nineteen patients received urokinase 200,000 IU/L in 1 L of Jonosteril, and six patients received 100,000 IU/L. The median irrigation rate was 48 mL/h (IQR 35–76). The median interval between the procedure and post-treatment imaging was 5 days (IQR 4–6).

### 3.2 Radiologic findings

Paired imaging measurements were available for 25 patients. The two patients with rebleeding were included in this analysis; their selected CT examinations preceded rescue craniotomy and were obtained before completion of the planned treatment course.

Median hematoma volume decreased from 83.8 cm^3^ (IQR 62.4–111.0) before treatment to 21.0 cm^3^ (IQR 5.0–35.6) on the selected follow-up CT (p < 0.0001).

The median percentage volume reduction was 75.0% (IQR 63.0–93.1) (Fig. 2A). Median midline shift decreased from 6.0 mm (IQR 3.5–8.5) to 0.0 mm (IQR 0.0–2.0) (p < 0.0001) (Fig. 2B). Median hematoma thickness decreased from 12.0 mm (IQR 9.0–15.0) to 4.0 mm (IQR 2.0–6.0) (p < 0.0001).

**Fig. 2:**
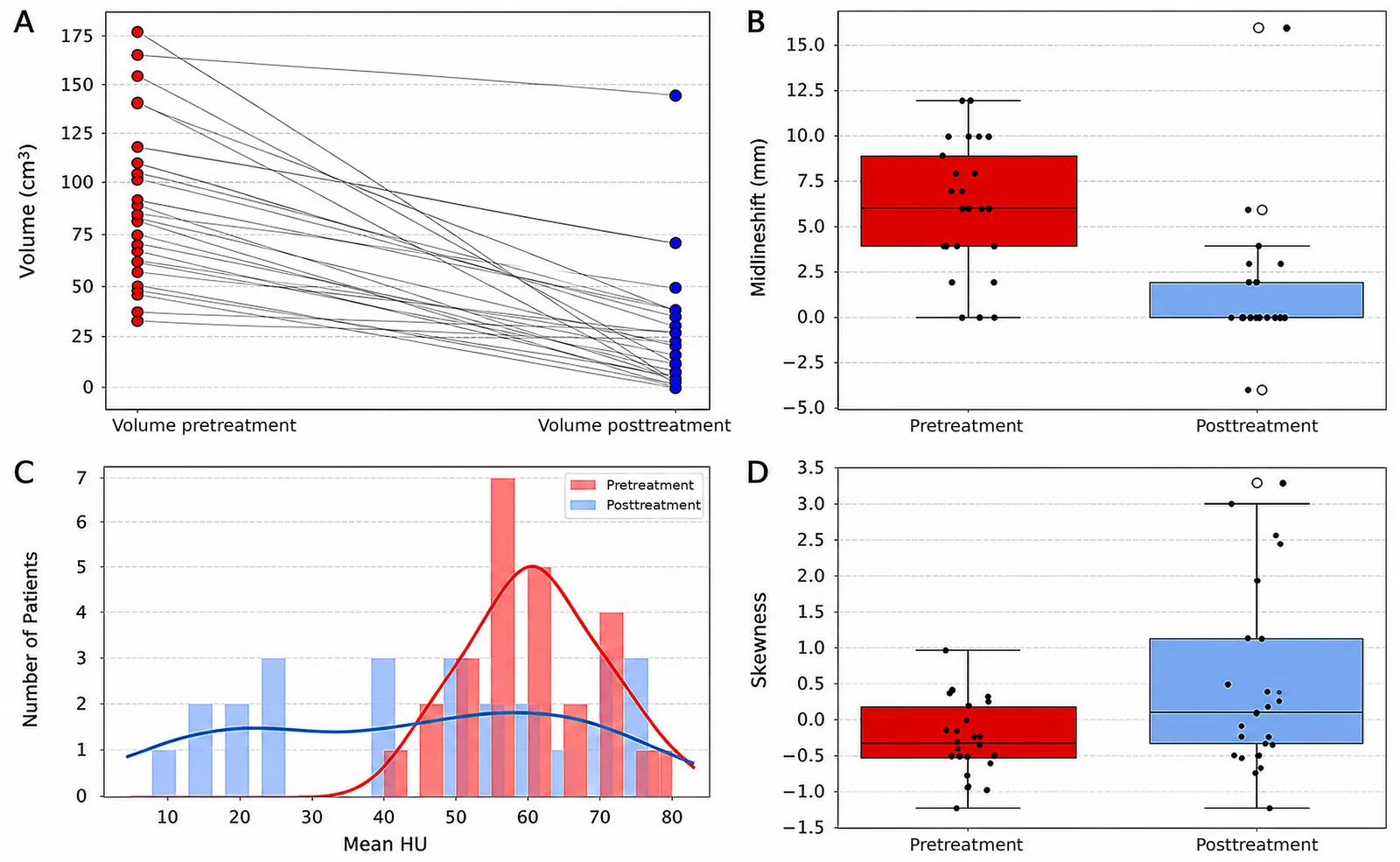
Radiologic characteristics of acute subdural hematomas before and after treatment. (A) Individual hematoma volumes before and after treatment. Among 25 patients with complete paired measurements, median hematoma volume decreased from 83.8 cm^3^ to 21.0 cm^3^ (*p* < 0.0001). The median percentage volume reduction was 75.0%. (B) Midline shift and hematoma thickness before and after treatment. Median midline shift decreased from 6.0 mm to 0.0 mm (*p* < 0.0001), with a corresponding reduction in hematoma thickness. (C) Median hematoma radiodensity decreased from 59.9 Hounsfield Units (HU) to 48.0 HU (*p* = 0.0025). (D) Skewness of the hematoma attenuation distribution changed from −0.56 to −0.14 (*p* = 0.0203). Filled circles represent individual patient values; open circles indicate outliers.

The median hematoma surface area decreased from 28.5 cm^2^ (IQR 21.8–35.2) to 9.7 cm^2^ (IQR 5.3–17.7) (p < 0.0001). Median radiodensity decreased from 59.9 HU (IQR 55.0–64.8) to 48.0 HU (IQR 32.0–72.0) (p = 0.0025) (Fig. 2C).

Skewness changed from −0.56 (−0.77 to −0.03) to −0.14 (−0.63 to 0.97) (p = 0.0203) (Fig. 2D). Kurtosis did not differ significantly (3.46 [2.85 to 4.10] vs. 3.41 [2.52 to 4.79]; p = 0.61).

Absolute volume reduction correlated strongly with preoperative hematoma volume (Spearman's ρ = 0.81, p < 0.001). Percentage volume reduction was inversely correlated with preoperative median HU (ρ = −0.47, p = 0.018). No significant correlations were observed between percentage volume reduction and preoperative volume, age, irrigation duration, irrigation rate, or fibrinolytic dose (all p > 0.09).

The radiologic characteristics of acute subdural hematomas before and after treatment are shown in Fig. 2**.** Baseline radiomic features are summarized in Table 1.

### 3.3 Outcome

Given the small sample size and the multiple unadjusted comparisons performed, all analyses should be considered exploratory.

The median premorbid mRS score was 2 (IQR, 0–3), increasing to 3 (IQR, 2–4) at discharge. At the 3-month follow-up, the median mRS score was 2 (IQR, 1–3), and a favorable functional outcome, defined as an mRS score ≤ 3, was achieved in 72% of patients.

Five patients (20.0%) experienced procedure-related complications (Table 2). Their median premorbid, pretreatment, and discharge mRS scores were 3 (range, 0–4), 5 (range, 1–5), and 5 (range, 2–6), respectively. The median age-adjusted Charlson Comorbidity Index (aCCI) was 6 (range, 4–10).

**Table 2:** Complications following TDC-FIT, including timing, antithrombotic therapy, rescue treatment, and functional outcome at 3 months.

Event	Timing	Recorded antithrombotic therapy	Rescue treatment	mRS 3 months
**Rebleeding**	Before completion of TDC-FIT at day 1	Marcumar	Conversion to open craniotomy	3
**Rebleeding**	Before completion of TDC-FIT at day 3	antiplatele	Conversion to open craniotomy	6
**Symptomatic chronic SDH recurrence requiring readmission**	Later follow-up; interval 4 weeks	None	Repeat twist-drill craniostomy	0
**Symptomatic chronic SDH recurrence requiring readmission**	Later follow-up; interval 6 weeks	Marcumar + antiplatelet	Repeat twist-drill craniostomy	1
**Mechanical device failure: drainage-lumen occlusion**	Druring TDC-FIT	Marcumar	Flushing with normal saline and urokinase until patency was restored.	3

Rebleeding requiring conversion to open craniotomy occurred in two patients (8%). Both had a premorbid mRS score of 3 and a pretreatment mRS score of 5. One patient (premorbid aCCI, 6) subsequently died from sepsis, (final mRS score of 6.) The other patient (premorbid aCCI, 4) had an mRS score of 3 at the 3-month follow-up.

Symptomatic recurrence of chronic subdural hematoma (cSDH) requiring readmission occurred in two patients (8%). The recurrences were diagnosed 4 and 6 weeks after the initial treatment, respectively, and both patients underwent repeat twist-drill craniostomy. Both had a premorbid mRS score of 0 and a discharge mRS score of 2. At the 3-month follow-up, their mRS scores were 0 and 1, respectively, and their premorbid aCCI scores were 8 and 5.

Mechanical device failure due to occlusion of the drainage lumen occurred in one patient (4%). This patient had a premorbid aCCI score of 10 and a premorbid mRS score of 3. The mRS score remained 3 at pretreatment assessment, discharge, and the 3-month follow-up. No clinical sequelae attributable to the device malfunction were reported.

No significant associations were observed between 3-month follow-up mRS scores and preoperative hematoma volume, preoperative median Hounsfield unit (HU) values, or irrigation duration. Patients with a hematoma volume reduction ≥50% (n = 22, 88%) had a lower median mRS score at 3-month follow-up than those with a reduction <50% (n = 3, 12%) (2 vs. 4; p = 0.034).

Higher irrigation rates were associated with lower 3-month follow-up mRS scores (Spearman's ρ = −0.51, p = 0.008). Similarly, higher cumulative fibrinolytic doses were associated with lower mRS scores (ρ = −0.48, p = 0.014). Lower postoperative HU values showed a trend toward an association with better functional outcomes (p = 0.06; Fig. 3).

**Fig. 3:**
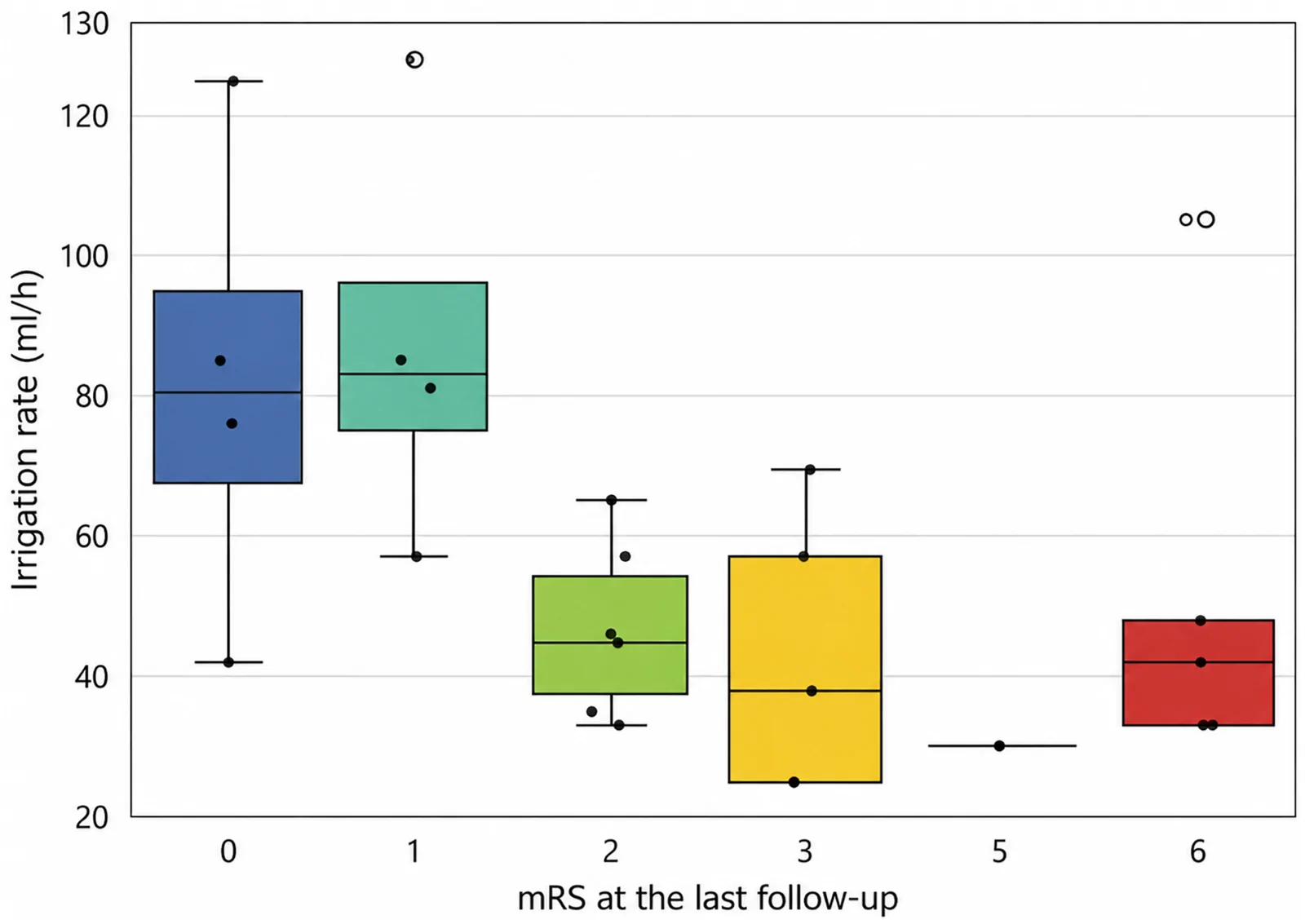
Box plots of irrigation rate (mL/h) according to modified Rankin Scale (mRS) score at the last follow-up. Each box represents the distribution of irrigation rates within the corresponding mRS category, with individual patient values overlaid. Higher irrigation rates were generally observed in patients with better functional outcomes (mRS 0–1), whereas lower irrigation rates were more common in patients with poorer outcomes (mRS ≥2, particularly mRS 5–6). Filled circles represent individual patient values, and open circles indicate outliers.

No statistically significant associations were identified between rebleeding or recurrence and irrigation duration, irrigation rate, or cumulative fibrinolytic dose (all p > 0.05).

## 4 Discussion

In neurologically stable patients with acute subdural hematoma, TDC-FIT may represent a minimally invasive alternative to open craniotomy, with treatment being associated with substantial hematoma volume reduction and imaging findings potentially consistent with clot liquefaction.

Median hematoma volume decreased from 83.8 cm^3^ to 21.0 cm^3^, corresponding to a median reduction of 75% (p < 0.0001). Median hematoma thickness decreased from 12 mm to 4 mm (p < 0.0001), and midline shift was effectively resolved in most patients (p < 0.0001). These findings indicate that TDC-FIT can achieve marked hematoma evacuation in neurologically stable patients. By facilitating minimally invasive clot evacuation, TDC-FIT may reduce the risk of procedure-related complications associated with open craniotomy, including brain contusion, surgical site infections, cerebrospinal fluid fistulas, and anesthesia-related adverse events. Moreover, pressure-controlled hematoma evacuation may hypothetically reduce abrupt intracranial pressure shifts and secondary brain injury. However, the temporal dynamics of hematoma evacuation were not assessed in this study, and these effects remain speculative. Further controlled studies are needed to evaluate evacuation kinetics and their clinical relevance.

Radiomic analysis revealed changes in hematoma characteristics following TDC-FIT. Median hematoma radiodensity decreased from 59.9 HU to 48.0 HU, while skewness shifted from −0.56 to −0.14. These findings may reflect changes in hematoma composition and are potentially consistent with clot liquefaction. However, they cannot be interpreted as direct evidence of fibrinolysis, as alternative explanations include dilution by irrigation fluid, partial-volume effects, and variability in image segmentation. Mechanistically, irrigation may facilitate hematoma clearance by disrupting fibrin networks and promoting the removal of cellular debris, potentially favoring the transition from a more solid to a more fluid hematoma consistency.

Experimental and preclinical studies indicate that blood in the subdural space can trigger neuroinflammatory responses and compromise blood–brain barrier integrity (Jussen et al., 2017). In animal models, subdural infusion of whole blood has been shown to increase inflammatory markers and blood–brain barrier leakage compared with plasma alone, suggesting that blood components themselves can induce secondary tissue injury. Inflammation is also implicated in the development of chronic subdural hematomas, as dural border cell injury and the persistent presence of blood products stimulate cytokine release (e.g., TNF-α, IL-1, IL-6) and membrane formation, potentially promoting fluid accumulation and hematoma progression (Jussen et al., 2017; Suzuki et al., 1998; Frati et al., 2004). However, the direct translation of these mechanisms to humans treated with TDC-FIT remains speculative.

The observed association between higher irrigation rate and fibrinolytic dose and improved functional outcomes (Spearman's ρ ≈ −0.51 for irrigation rate, p = 0.008; ρ ≈ −0.48 for fibrinolytic dose, p = 0.014) may reflect more effective clearance of proinflammatory and cytotoxic blood breakdown products. This is further supported by the inverse relationship between percentage volume reduction and preoperative median HU, potentially suggesting an association between preoperative hematoma attenuation, hematoma volume reduction, and irrigation intensity.

However, the achieved irrigation rate was likely not independent of hematoma characteristics or intracranial conditions. In particular, technical and physiological factors—such as hematoma viscosity, septations, compartmentalization, and intracranial pressure dynamics—may have influenced the feasible irrigation intensity. Patients in whom higher irrigation rates could be safely maintained may therefore represent a subgroup with more favorable hematoma morphology and drainage conditions, whereas more complex or compartmentalized hematomas may have limited achievable flow rates and are also potentially associated with worse functional outcomes. These findings should be interpreted as exploratory associations rather than evidence of causality.

In contrast to its use in chronic subdural hematomas, application of TDC-FIT in acute subdural hematoma in our cohort was associated with an 8% rebleeding rate requiring conversion to open craniotomy, underscoring the need to better understand the underlying mechanisms of rebleeding, identify patients at increased risk, and incorporate these considerations into future indication algorithms (Doria-Medina et al., 2025; Bissolo et al., 2026).

Patient selection remains a major challenge in the application of TDC-FIT. The aim is to identify patients who require active treatment but whose clinical condition does not necessitate immediate emergency decompression. In selected patients, particularly older individuals with focal neurological symptoms and substantial radiological mass effect but relatively preserved consciousness, minimally invasive and gradual hematoma evacuation may be considered following careful clinical assessment and confirmation of neurological and radiological stability. Nevertheless, conventional recommendations support surgical evacuation of acute traumatic SDH exceeding 10 mm in thickness or 5 mm in midline shift, regardless of GCS. TDC-FIT should therefore not be considered a substitute for urgent surgery when indicated. The retrospective nature of patient selection and the absence of standardized quantification of focal neurological deficits limit the generalizability of our findings and highlight the need for clearer selection criteria.

This study has additional limitations, including its retrospective, single-center design, small sample size, and absence of a control group. The latter precludes direct comparison with conventional surgical or conservative management and prevents conclusions regarding the comparative efficacy or clinical superiority of TDC-FIT. Identifying an appropriate comparator population remains a key challenge for future prospective studies, given differences in baseline hematoma characteristics, clinical presentation, and treatment indications. Furthermore, correlation analyses were exploratory, without adjustment for multiple comparisons or multivariable modeling, and residual confounding cannot be excluded. Volumetric and radiomic measurements were based on semi-automated CT segmentation, which may introduce measurement variability despite the use of a standardized imaging protocol on a single scanner.

Strengths include systematic volumetric and radiomic assessment and a clearly defined treatment regimen with prospectively recorded irrigation parameters and fibrinolytic dosing, supporting the consistency of the data and their value for hypothesis generation.

Overall, TDC-FIT was associated with substantial hematoma reduction in selected neurologically stable patients with aSDH, accompanied by radiomic changes potentially consistent with clot transformation. The observed associations with functional outcomes are exploratory and do not establish causality. Prospective, controlled studies are needed to define appropriate patient-selection criteria, establish safety and efficacy relative to existing treatment strategies, and determine optimal treatment parameters.

## 5 Conclusion

TDC-FIT substantially reduced hematoma volume and midline shift in patients with aSDH who did not require immediate craniotomy, consistent with substantial hematoma evacuation and CT attenuation changes potentially reflecting alterations in hematoma composition.

However, given the retrospective design and limited sample size, these findings should be considered preliminary and hypothesis-generating and need confirmation in larger prospective studies.

## Disclosures

In Europe, intrathecal urokinase use is off-label. Intrathecal infusion using the LiquoGuard 7 system is approved only for lumbar infusion testing within the manufacturer's intended use. Accordingly, the application of the LiquoGuard 7 system described in this paper represents off-label use.

## Declaration of competing interest

The authors declare the following financial interests/personal relationships which may be considered as potential competing interests: Marco Bissolo reports article publishing charges was provided by University of Freiburg Department of Neurosurgery. Marco Bissolo reports a relationship with University of Freiburg Department of Neurosurgery that includes: non-financial support. Marco Bissolo has patent pending to NO. If there are other authors, they declare that they have no known competing financial interests or personal relationships that could have appeared to influence the work reported in this paper.
